# Association of thyroid cancer with human papillomavirus infections

**DOI:** 10.1038/s41598-023-49123-z

**Published:** 2024-01-03

**Authors:** Tzong-Hann Yang, Shih-Han Hung, Yen-Fu Cheng, Chin-Shyan Chen, Herng-Ching Lin

**Affiliations:** 1 Department of Otorhinolaryngology, Taipei City Hospital, Taipei, Taiwan; 2 Department of Speech, Language and Audiology, National Taipei University of Nursing and Health, Taipei, Taiwan; 3 Department of Otolaryngology-Head and Neck Surgery, School of Medicine, National Yang Ming Chiao Tung University, Taipei, Taiwan; 4 Center of General Education, University of Taipei, Taipei, Taiwan; 5 Research Center of Data Science on Healthcare Industry, College of Management, Taipei Medical University, Taipei, Taiwan; 6 Department of Otolaryngology, School of Medicine, Taipei Medical University, Taipei, 110 Taiwan; 7 Department of Otolaryngology, Wan Fang Hospital, Taipei, 110 Taiwan; 8 International Ph.D. Program in Medicine, College of Medicine, Taipei Medical University, Taipei, 110 Taiwan; 9 Department of Medical Research, Taipei Veterans General Hospital, Taipei, Taiwan; 10 Department of Otolaryngology-Head and Neck Surgery, Taipei Veterans General Hospital, Taipei, Taiwan; 11 Department of Economics, National Taipei University, New Taipei City, Taiwan; 12 School of Health Care Administration, College of Management, Taipei Medical University, Taipei, Taiwan; 13 Research Center of Sleep Medicine, Taipei Medical University Hospital, Taipei, Taiwan

**Keywords:** Cancer, Endocrinology, Risk factors

## Abstract

While Human Papillomavirus (HPV) particles have been detected in a small proportion of benign thyroid nodules or thyroid cancer cases, a role of HPV in these thyroid conditions has not been established. This study aims to investigate the association of HPV infection with thyroid cancer (TC) using a nationwide population-based study. We retrieved data for this case–control study from Taiwan's Longitudinal Health Insurance Database 2010. The study sample included 3062 patients with TC and 9186 propensity-scored matched controls. We employed multivariate logistic regression models to quantitatively evaluate the association of TC with HPV infections after taking age, sex, monthly income, geographic location and urbanization level of the patient's residence, diabetes, hypertension, and hyperlipidemia into considerations. Chi-squared test revealed that there was a significant difference in the prevalence of prior HPV infections between patients with TC and controls (15.3% vs. 7.6%, *p* < 0.001). The adjusted odds ratio of prior HPV infections for patients with TC was 2.199 (95% CI = 1.939–2.492) relative to controls. The adjusted ORs of prior HPV infections for patients with TC was similar for males and females. Our research suggests a significant link between HPV infection and the development of TC.

## Introduction

The observed increase in thyroid cancer diagnoses over the past few decades has prompted the scientific community to search for potential causes of this trend. In the United States, for instance, the incidence of thyroid cancer has tripled in the past 30 years, with about 45,000 new cases being diagnosed annually^1,2^. As the most common endocrine cancer, thyroid cancer can disrupt vital bodily functions regulated by the thyroid gland, which includes breathing, heart rate, nervous system, body temperature, and more^3,4^.

Although thyroid cancer is largely treatable, identifying and preventing its cause could help minimize the number of patients experiencing symptoms and the side effects of treatment. The exact cause of thyroid cancer still remains unexplored, with identified risk factors ranging from inherited genetic conditions and lifestyle habits like smoking and obesity to environmental exposure to nonmedical radiation^5,6^.

The association between viral infection and the development of thyroid cancer remains largely unknown. Human papillomavirus is a virus associated with several cancer types, and high-risk human papillomavirus have been linked to about 3% of cancers in women and 2% of cancers in men, including cancers of the cervix, anus, genitals, and throat^7,8^. The presence of DNA from certain viruses, including the human papillomavirus, has been detected in thyroid tissues and tumors^9^. While human papillomavirus particles have been detected in a small proportion of benign thyroid nodules, thyroid dysfunction, or thyroid cancer cases, a definitive causative role of HPV in these thyroid conditions has not been established^10,11^.

As thyroid cancer continues to rise among populations, identifying previously unexplored etiologies of the disease becomes mandatory. As the role of human papillomavirus in the development of thyroid cancer presents an intriguing possibility, this study aims to investigate the association of human papillomavirus infection with the development of thyroid cancer using a nationwide population-based study.

## Methods

### Database

We retrieved data for this case–control study from Taiwan's Longitudinal Health Insurance Database 2005. Taiwan introduced its single-payer compulsory social healthcare insurance program in 1995, this system's claims data are released as the National Health Insurance Research Database. The Taiwan National Health Insurance program provides low-copayment and comprehensive medical care for all Taiwanese citizens. The National Health Insurance Research Database consists of de-identified registry for beneficiaries, ambulatory care claims, inpatient claims, prescriptions dispensed at pharmacies, registry for medical facilities, and registry for board-certified specialists that are primarily used for reimbursement purposes. The Data Science Centre of the Ministry of Health and Welfare of Taiwan have created a number of sub-datasets within the National Health Insurance Research Database, including the Longitudinal Health Insurance Database 2005. The Longitudinal Health Insurance Database 2005 includes basic demographic information, disease diagnoses, prescriptions, and operations of a random sample of 2,000,000 National Health Insurance beneficiaries. Many researchers and scholars have employed this large Longitudinal Health Insurance Database 2005 to carry out biomedical studies of diseases and treatments.

The study was approved by the Research Ethics Committee of National Taiwan University (202012EM075). The Data Science Centre of the Ministry of Health and Welfare of Taiwan has encrypted the names of patients, health care providers, and medical institutions with unique and anonymous identifiers in order to protect the privacy of patients. Theerfore, the Longitudinal Health Insurance Database 2005 is a deidentified administrative dataset, so patient informed consent was waived by the Research Ethics Committee of National Taiwan University in this study.

### Identification of study patients

This study was designed as a case–control study. As for selection of cases, we identified 3062 patients aged 20 years and above and who had received a first-time diagnosis of thyroid cancer (ICD-9-CM 193, ICD-10-CM code C73) between January 1, 2012 and December 31, 2019. All medical facilities used the International Classification of Diseases, Ninth Edition, Clinical Modification (ICD-9-CM) to record diagnosis in the Longitudinal Health Insurance Database 2010 before 2016. The ICD-10-CM system has been used since 2016. In addition, we assured that only patients with at least two different medical claims showing a diagnosis of thyroid cancer by board-certified oncologists were included in this study because of potential concerns about the validity of diagnosis coding. In Taiwan, under the National Health Insurance program, if a patient was suspected of having thyroid cancer, the physician would make a provisional diagnosis of thyroid cancer to perform further clinical or lab tests including thyroid function blood tests, ultrasound imaging and biopsy test for confirmation. This was done to prevent receiving possible fines for performing unnecessary or inappropriate procedures. Therefore, to assure the validity of the thyroid cancer diagnosis, patients were only selected if they had received two different medical claims showing a diagnosis of thyroid cancer. Cross-referencing with the Taiwan Cancer Registry also provided diagnostic confirmation. We further defined the date of receiving their first-time thyroid cancer diagnosis as the index date in order to ensure that the selected patients were newly diagnosed cases.

This study attempeted to examine the association of thyroid cancer with human papillomavirus infections, we therefore employed propensity-score-matching method to select the controls from the remaining beneficiaries aged 20 years and above from the Registry of beneficiaries of Longitudinal Health Insurance Database 2010. Propensity score matching is a quasi-experimental method that allows us to construct an artificial control group by matching each patient with thyroid cancer with a non-thyroid cancer beneficiary of similar characteristics. All enrollees who had a history of thyroid cancer in a medical claim were excluded. Propensity scores were calculated for all selected 9186 patients with thyroid cancer and remaining beneficiaries using the logistic regression model with adjustment for age, sex, monthly income (NT)$0–15,840, NT$15,841–25,000, ≥ NT$25,001; US$1 ≈ NT$28 in 2021), geographic location (Northern, Central, Southern, and Eastern) and urbanization level of the patient's residence (5 levels, 1 most urbanized, 5 least urbanized) and diabetes, hypertension, and hyperlipidemia. Finally, each sampled patient with thyroid cancer was matched for three controls without thyroid cancer through the nearest neighbor random matching algorithm with caliper adjustment, using a priori value for the calipers of ± 0.01. While for cases, we assigned the year of the index date as the year in which the cases received their first thyroid cancer diagnosis, for controls, the year of the index date was simply a matched year in which controls had an ambulatory care visit. As a result, the study sample was composed of 3062 patients with thyroid cancer and 9186 controls without thyroid cancer.

### Measures of outcomes

We identified human papillomavirus infections cases based on ICD-9-CM codes078.10, 078.11, 078.12, 078.19, 795.1, and 079.4, along with records of positive polymerase chain reaction test results or ICD-10-CM codes B07.08, B97.7, R85.81, R85.82, R87.82 and A63. This study only included human papillomavirus infections cases if they had received at least one diagnoses of human papillomavirus infections prior to the index date.

### Statistical analysis

All statistical analyses were performed using the SAS system (SAS System for Windows, vers. 9.4, SAS Institute, Cary, NC). We used chi-square tests and t-tests to explore differences in baseline characteristics between patients with thyroid cancer and the controls. Furthermore, we employed multivariate logistic regression models to quantitatively evaluate the association of thyroid cancer with human papillomavirus infections after taking age, sex, monthly income, geographic location and urbanization level of the patient's residence, diabetes, hypertension, and hyperlipidemia into considerations. Estimated odds ratio (OR) and their 95% confidence intervals (CIs) were used to quantify the difference in odds of human papillomavirus infections between patients with thyroid cancer and the controls. All *p*-values were two-sided, and a *p*-value < 0.05 was considered statistically significant.

### Institutional review board statement

The study obtained approval from the Research Ethics Committee of National Taiwan University (202012EM075) and is compliant with the Declaration of Helsinki.

### Informed consent statement

Patient consent was waived because this study used administrative data.

## Results

Of the 12,248 sampled patients, the mean age for the study sample was 54.5 ± 13.8 years (± standard deviation). After using propensity-scored matching, Table 1 shows that there were no statistically significant differences between patients with thyroid cancer and controls in terms of sex (*p* > 0.999), urbanization level (*p* > 0.999), monthly income (*p* = 0.939), and geographic region (*p* = 0.951). In addition, there were no statistically significant differences in the prevalence of diabetes (26.1% in both groups, *p* > 0.999), hypertension (42.0% in both groups, *p* > 0.999) and hyperlipidemia (43.45% in both groups, *p* > 0.999) between patients with thyroid cancer and controls.

**Table 1:** Demographic characteristics of patients with thyroid cancer and propensity score-matched controls (n = 12,248).

Variable	Patients with thyroid cancer (*n* = 3062)		Propensity score-matched controls (*n* = 9186)		*p* value
	Total no	%	Total no	%	
Age, mean (SD)	54.5 (13.8)		54.5 (13.8)		0.975
Males	755	13.8	2265	13.8	> 0.999
Monthly income					0.939
< NT$1–15,841	479	15.6	1416	15.4	
NT$15,841–25,000	1003	32.8	3033	33.0	
≥ NT$25,001	1580	51.6	4734	51.6	
Geographic region					0.951
Northern	1531	50.0	4623	50.3	
Central	613	20.0	1825	19.9	
Southern	863	28.2	2586	28.2	
Eastern	55	1.8	152	1.6	
Urbanization level					> 0.999
1 (most urbanized)	925	30.2	2766	30.1	
2	951	31.1	2853	31.1	
3	456	14.9	1374	15.0	
4	345	11.3	1033	11.2	
5 (least urbanized)	385	12.6	1160	12.6	
Hypertension	1287	42.0	3861	42.0	> 0.999
Hyperlipidemia	1329	43.4	3987	43.4	> 0.999
Diabetes	800	26.1	2400	26.1	> 0.999

Table 2 shows the prevalence of prior human papillomavirus infections among the sampled patients. There were 1171 (9.6%) out of 12,248 sampled patients had HPV infections prior to the index date. Chi-squared test revealed that there was a significant difference in the prevalence of prior human papillomavirus infections between patients with thyroid cancer and controls (15.3% vs. 7.6%, *p* < 0.001).

**Table 2:** Prevalence rates of HPV among patients with thyroid cancer vs. controls.

Presence of HPV	Total (*n* = 12,248)		Patients with thyroid cancer (*n* = 3062)		Controls (*n* = 9186)	
			*n*, %			
Yes	1171	9.6	469	15.3	702	7.6
No	11,077	90.4	2593	84.7	8484	92.4
Odds ratio^a^ (95% CI)	–		2.199* (1.939–2.492)		1.000	

^a^Adjusted for age, sex, monthly income, geographic region, urbanization level, diabetes, hypertension, and hyperlipidemia; *p < 0.001.

Table 2 also shows the results of multivariate logistic regression analyses. The OR of prior human papillomavirus infections for patients with thyroid cancer was 2.199 (95% CI = 1.939–2.492, *p* < 0.001) relative to controls after adjusting for monthly income, geographic location, urbanization level of residence, diabetes, hypertension, and hyperlipidemia.

Table 3 presents the adjusted ORs of prior human papillomavirus infections among patients with thyroid cancer vs. controls according to sex. Of the female sampled patients, the adjusted OR of prior human papillomavirus infections for patients with thyroid cancer was 2.116 (95% CI = 1.830–2.446) compared to controls. Similarly, a statistically significant association between prior human papillomavirus infections and thyroid cancer was still observed in male sampled patients (adjusted OR = 2.480, 95% confidence interval = 1.919–3.189).

**Table 3:** Prevalence rates of HPV among patients with thyroid cancer vs. controls according to sex.

Males						
Presence of HPV	Total (*n* = 3020)		Patients with thyroid cancer (*n* = 755)		Controls (*n* = 2265)	
Yes	289	9.6	123	16.3	166	7.3
No	2731	90.4	632	83.7	2099	92.7
Odds ratio^a^ (95% CI)	–		2.480* (1.929–3.189)		1.000	

Females						
Presence of HPV	Total (*n* = 9228)		Patients with thyroid cancer (*n* = 2307)		Controls (*n* = 6923)	
Yes	882	9.6	346	15.0	538	7.7
No	8346	90.4	1961	85.0	6385	92.3
Odds ratio^a^ (95% CI)			2.116* (1.830–2.446)		1.000	

^a^Adjusted for age, sex, monthly income, geographic region, urbanization level, diabetes, hypertension, and hyperlipidemia; *p < 0.001.

## Discussion

Our research discovered a link between human papillomavirus infections and the onset of thyroid cancer. After accounting for other influential factors like income, location, urbanization level, and pre-existing conditions such as diabetes, hypertension, and hyperlipidemia, those diagnosed with thyroid cancer were 2.199 times more likely to have had a previous human papillomavirus infection in comparison to the controls. This association was similar in males and females.

The recent population-based study provides an important overview of international trends in thyroid cancer incidence, demonstrating a significant increase over the past three decades, predominantly driven by the papillary thyroid cancer subtype^12^. The incidence and incidence-based mortality also increased, suggesting an actual increase in clinically significant disease^12–14^. The global burden of thyroid cancer has been reported to be increasing, with almost half (41.73% for incidence, 50.92% for deaths, and 54.39% for disability-adjusted life-years) of the thyroid cancer burden reported to be in Southern and Eastern Asia^15^.

The etiology of thyroid cancer is multifactorial and complex, involving both genetic and environmental factors. Research has identified several genetic mutations, such as those found in the BRAF, RAS, and RET/PTC genes, which are often associated with various types of thyroid cancer^16^. These mutations can result in uncontrolled cell growth and division, leading to tumor formation^17^. Environmental factors, such as exposure to ionizing radiation, especially during childhood, also significantly increase the risk of thyroid cancer^6^. Additionally, nutritional status, particularly iodine deficiency or excess, has been implicated in developing this disease^18^. More recent evidence suggested that the immune microenvironment associated with thyroid cancer may play a critical role in tumor invasion^19^. Interestingly, little has been addressed regarding the possible role of human papillomavirus infections in developing thyroid cancer.

Human papillomavirus is a sexually transmitted infectious agent that plays a significant role in the etiology of various cancers. The most notable of these is cervical cancer, which is almost entirely attributable to human papillomavirus infection. Beyond the cervix, HPV is also implicated in many other anogenital cancers (such as vaginal, vulvar, penial, and anal) and oropharyngeal cancers. human papillomavirus’s role in carcinogenesis largely stems from the actions of its viral oncoproteins E6 and E7, which induce degradation of the tumor suppressor proteins p53 and pRb, respectively. This degradation disrupts normal cell cycle control and promotes progression into the S phase of the cell cycle without the usual G1 arrest, leading to uncontrolled cellular proliferation. Furthermore, these viral oncoproteins are known to interfere with various cellular processes, including DNA repair, angiogenesis, and apoptosis, contributing to further genomic instability and the eventual development of cancer^20,21^.

Recently, a study by Dialameh et al. investigated the possible role of human papillomavirus in papillary thyroid carcinoma by retrospectively analyzing the thyroid tissue specimens for the HPV through polymerase chain reaction (PCR) and found that the HPV PCR positivity was observed in 3.8% of benign thyroid nodules and 13.4% of papillary thyroid carcinoma samples, and the prevalence of human papillomavirus PCR positivity in the papillary thyroid carcinoma tissues was significantly more than the benign thyroid nodules^9^. On the contrary, a study also using the PCR in investigating the presence of human papillomavirus DNA in nodular thyroid diseases has revealed that human papillomavirus DNA was not detected in either nodular or normal thyroid tissue^22^. A study by Mostafaei et al. investigated further the simultaneous effects of human papilloma and Epstein-Barr virus viral factors on the progression of breast and thyroid Cancer. The study demonstrated a significant association between EBV and human papillomavirus—genes, anoikis resistance, and the development of breast and thyroid cancers^10^. These molecular-level findings might provide some evidence supporting our findings based on the large-scale population database.

While human papillomavirus DNA has been found in thyroid tissues and tumors in previous studies, suggesting a potential connection to thyroid cancer, a role for HPV has yet to be established. It should be noted that not only human papillomavirus has been reported to be associated with thyroid cancer. A systemic review and meta-analysis have demonstrated that the highest associations were observed between thyroid cancer risk and Simian Vacuolating Virus 40 (SV40) and B19 infections, and the lowest non-significant association was found between thyroid cancer risk and Poliovirus type 1 infection with a significant heterogeneity observed between included studies^11^.

The observation that Human Papillomavirus (HPV) has comparable prevalence rates in both male and female control groups, coupled with the finding that thyroid cancer is more commonly seen in females, underscores the necessity for additional research. This research should aim to unravel the distinct mechanisms of HPV-induced carcinogenesis in males and females. Given the disparity in thyroid cancer rates despite similar HPV infection rates, it is imperative to explore gender-specific biological, genetic, and lifestyle factors that might influence the progression from HPV infection to the development of thyroid cancer. This investigation is crucial in enhancing our understanding of gender differences in cancer development and tailoring more effective prevention and treatment strategies.

The value of human papillomavirus vaccination regarding cancer prevention should also be mentioned in the interpretation of our study result. The effectiveness of prophylactic human papillomavirus vaccines in preventing infection and diseases such as cervical, vulvovaginal, and anal diseases associated with specific human papillomavirus types has been confirmed by both high-quality clinical trials and population-based studies. These vaccines employ virus-like particles devoid of the viral genome to stimulate strong neutralising antibody responses, though they do not enhance the clearance of existing infections, underscoring the importance of early vaccination. Despite the confirmed efficacy of these vaccines in preventing human papillomavirus-related cancers and anogenital warts, challenges remain in improving vaccine coverage, especially among low-income and marginalized populations^23^.

Based on our result that the incidence of developing thyroid cancer is elevated in both human papillomavirus-infected males and females, gender-neutral vaccination policies, including boys, might also be necessary. A study in China demonstrated that the rate of increasing thyroid cancer incidence trend was lower in older age groups, implying the increased incidence of thyroid cancer is mainly in the younger age group, which would most likely benefit from the vaccination^24^. However, as the incidence is relatively lower when compared to other well-known human papillomavirus-related cancers, such as oropharyngeal cancer and laryngeal cancer, the effectiveness of large-scale human papillomavirus vaccination in preventing thyroid cancer is expected to be limited.

The results of this study could have several potential clinical applications. Firstly, it may be advisable to monitor individuals with a history of human papillomavirus infection for thyroid cancer. Secondly, the study results highlight the need to raise awareness about human papillomavirus-related risks and promote preventive strategies in both men and women. If further research confirms the link between human papillomavirus and thyroid cancer, human papillomavirus vaccinations could possibly be used as a preventive measure. Lastly, targeted therapies against human papillomavirus might be explored as a treatment approach for thyroid cancer.

Despite the comprehensive approach, this study has several limitations that should be considered. Firstly, the use of data from Taiwan's Longitudinal Health Insurance Database 2010 limits the generalizability of the findings to other regions and healthcare systems. While the Taiwanese healthcare system is robust and widely utilized, its cultural, demographic, and healthcare policy nuances may not represent other regions globally. Secondly, the study's retrospective nature inherently carries the potential for recall and selection bias. Identifying patients based on diagnostic codes might lead to misclassification errors due to inaccuracies in the documentation or coding processes. Thirdly, the study only considers human papillomavirus cases diagnosed before the index date, possibly overlooking undiagnosed cases at the time of thyroid cancer diagnosis, potentially leading to an underestimation of the relationship. The absence of latency information, except the precedence of HPV over thyroid cancer, means the study doesn't account for the possible long duration before a thyroid cancer diagnosis, making temporality of association unclear. Lastly, while the study accounted for some key confounders, such as age, sex, monthly income, geographic location, urbanization level, diabetes, hypertension, and hyperlipidemia, there could still be unmeasured confounding factors influencing the results, including obesity, somking status, dietary habits, family history of thyroid cancer and previous radiation exposure to the neck by medical and dental diagnostics.

More research, including prospective cohort and experimental studies, is needed to validate our findings and establish a causal relationship between human papillomavirus and thyroid cancer. Clinically, considering human papillomavirus history in thyroid cancer risk assessment, especially in men, may be beneficial. If the association is confirmed, human papillomavirus vaccination policies could be considered to include the possible protection against thyroid cancer.

In summary, our research suggests a significant link between human papillomavirus infection and the development of thyroid cancer. While further research is needed to corroborate this association and understand the underlying mechanisms, our study emphasizes the potential role of human papillomavirus in the development of thyroid cancer and the clinical implications thereof. This study supports the necessity for clinicians, researchers, and public health practitioners to be aware of the possible connection between human papillomavirus and thyroid cancer.

## Data Availability

Data from the National Health Insurance Research Database, now managed by the Health and Welfare Data Science Center (HWDC), can be obtained by interested researchers through a formal application process addressed to the HWDC, Department of Statistics, Ministry of Health and Welfare, Taiwan (https://dep.mohw.gov.tw/DOS/lp-2506-113.html. 02/01/2022).
